# Effects of Aging Biodegradable Agricultural Films on Soil Physicochemical Properties and Heavy Metal Speciation

**DOI:** 10.3390/toxics13040245

**Published:** 2025-03-26

**Authors:** Hao Wu, Tianmu Peng, Xueya Li, Yang Zhao, Fengshuo Huang, Peng Guo, Mingfu Lyu, Junhua Yin, Qin Liu, Shaban Gouda, Ibrahim Mohamed, Qing Huang, Xu Wang

**Affiliations:** 1 Key Laboratory of Agro-Forestry Environmental Processes and Ecological Regulation of Hainan Province/Hainan Provincial Academician Team Innovation Center/International Joint Research Center for the Control and Prevention of Environmental Pollution on Tropical Islands of Hainan Province/School of Environment Science and Engineering/Haide Residential College, Hainan University, Haikou 570228, China; wh17886735868@163.com (H.W.); ptm0905@163.com (T.P.); 22210830000014@hainanu.edu.cn (X.L.); zhaoyang1376@163.com (Y.Z.); fengshuo20210305@163.com (F.H.); huangqing@hainanu.edu.cn (Q.H.); 2 SINOPEC (Beijing) Research Institute of Chemical Industry Co., Ltd., Beijing 100013, China; guopeng.bjhy@sinopec.com (P.G.); lumf.bjhy@sinopec.com (M.L.); 3 Shandong Qingtian Plastic Co., Ltd., Zibo 255410, China; yjh13668630343@163.com; 4 Institute of Environment and Sustainable Development in Agriculture, Chinese Academy of Agricultural Sciences, Beijing 100081, China; liuqin02@caas.cn; 5 Agricultural and Biosystems Engineering Department, Benha University, Banha 13511, Al-Qalyubia Governorate, Egypt; shaban.gouda@fagr.bu.edu.eg (S.G.); ibrahim.ali@fagr.bu.edu.eg (I.M.)

**Keywords:** biodegradable agricultural film, DOM, soil pH, enzyme activity, heavy metal speciation

## Abstract

Through soil incubation experiments, the effects of aged PBAT + PLA (polybutylene adipate terephthalate + polylactic acid) film fragments were analyzed. Surface characteristics and chemical structures of the films changed significantly after one (T2) and two years (T1) of aging compared to new films (T3). Both new and aged fragments reduced soil pH, altered enzyme activities, and influenced dissolved organic matter (DOM) fluorescence. Alkaline phosphatase activity declined by 33.2%, 23.8%, and 11.6% for T1, T2, and T3, respectively, while urease and sucrase activities increased in a time-dependent manner. The degree of soil humification rose by 66.4%, 60.4%, 49.3%, and 88.6% for T1, T2, T3, and T4, respectively, compared to the control (CK). Aged films exhibited stronger DOM fluorescence intensity than new films. Tessier extraction analysis revealed a decrease in exchangeable Cd by 22.9%, 13.1%, and 10.2% for T1, T2, and T3, respectively, while organically bound Cu increased. Correlation analysis indicated a significant positive relationship between soil humification and heavy metal bioavailability. These findings provide insight into the ecological effects of biodegradable agricultural films, offering a theoretical foundation for assessing their environmental risks and safety.

## 1. Introduction

Countries worldwide have become increasingly concerned about the serious pollution of soil caused by the large-scale production and use of plastics. The widespread use of agricultural films, which plays a crucial role in promoting increased crop yields and improving economic efficiency, has also raised environmental concerns [1]. By improving soil temperature and moisture conditions, reducing nutrient losses and effectively controlling weeds, agricultural film mulching technology significantly improves the efficiency of agricultural production and brings significant benefits to agricultural production. However, the most widely used agricultural film is mainly plastic film, and due to the high crystallinity of the polymer in plastic, its molecular arrangement is tightly ordered; this structure makes the intermolecular interaction force stronger and not easily destroyed by external factors, and its natural degradation often takes decades or even hundreds of years [2]. In addition to the improper recycling of agricultural films by farmers, residual agricultural films are left in the soil, and these residual plastic fragments undergo mechanical abrasion and a variety of degradation processes, such as biodegradation, photochemical decomposition, and oxidation [3,4,5]. Plastic debris is eventually converted into microplastics with a fragment size of less than 5 mm in the soil [6,7]. Research has shown that microplastics not only significantly affect soil physical and chemical properties and soil enzyme activities, but may also enter the human body through the food chain [8].

To cope with the environmental pollution caused by residual films, biodegradable agricultural films (PBAT + PLA) are gradually becoming an alternative to plastic agricultural films [9]. Although biodegradable agricultural films can theoretically decompose in the natural environment, the process of their complete degradation usually takes several years or even longer [10]. This means that during their life cycle, these biodegradable agricultural films will inevitably deteriorate in the physical environment under the influence of natural or anthropogenic factors, causing them to degrade under the action of factors such as oxidation, ultraviolet radiation, and thermal radiation [11,12]. It was shown that aged microplastics significantly enhanced the adsorption of metal ions compared to the original microplastics [13]. This suggests that the aging process not only changes the physicochemical properties of microplastics but also enhances their ability to adsorb other pollutants, thus affecting their behavior in the environment.

Microplastics likewise affect the physicochemical properties of soil, reduce soil pH 1.6%, and elevate soil urease 176% [14,15]; they also decrease soil alkaline phosphatase activity by releasing toxic substances due to their large specific surface area [16], and the photo-aging of microplastics promotes DOM release [17]. In turn, DOM affects the transport and transformation of soil heavy metals [18]. Although biodegradable agricultural films are a promising solution to plastic mulch pollution, their long-term interactions with soil, particularly the effects of aged on soil physicochemical properties and heavy metal speciation, remain poorly understood. These differences may further impact crop growth and soil health. Therefore, in this study, in-depth research was conducted on the effects of biodegradable agricultural films with aged soil properties and heavy metals, revealing their intrinsic mechanisms, to provide theoretical support for sustainable agricultural development and soil pollution management in the future.

Common biodegradable agricultural films (PBAT + PLA) currently available on the market were selected as the research objects, and their morphology and functional group changes were observed by various characterization methods, such as scanning electron microscopy (SEM) and Fourier transform infrared spectroscopy (FTIR); the effects of three different vintages of biodegradable agricultural film fragments on the soil pH, enzyme activity, and the chemical forms of soil DOM and heavy metals were determined by indoor soil incubation experiments. The objectives of the study were to (1) investigate the effect of naturally aged biodegradable agricultural film fragments on the chemical forms of Cd (Cadmium), Cu (Copper), Zn (Zinc), and Mn (Manganese) in soil; (2) elucidate the effect of biodegradable agricultural film on soil properties (pH, enzyme activity, DOM); and (3) explore the correlation between heavy metals and soil properties. This study provides critical evidence for understanding the environmental impacts of biodegradable agricultural films, aiming to establish a scientific basis for evaluating the ecological risks posed by the aging of these films in soil ecosystems.

## 2. Materials and Methods

### 2.1. Materials for Testing

Biodegradable agricultural films (composition of PBAT + PLA) from three different years, 2022, 2023, and 2024, were used. The test agricultural films were obtained from Sinopec Beijing Research Institute of Chemical Industry (BRICI), located in Beijing, China. The control treatment biomass was bagasse, which was obtained from the local market. Please state the name of the manufacturer, city, and country from where the equipment was sourced.

### 2.2. Residual Film Preparation and Characterization

Biodegradable agricultural films of three different aging periods were cut into 1 × 1 cm fragments to simulate soil residual film accumulation. Surface morphology changes before and after aging were analyzed using field emission scanning electron microscopy (SEM, Verios G4 UC, Thermo Fisher Scientific, Waltham, MA, USA), while functional group changes in the 500–4000 cm^−1^ region were characterized by Fourier transform infrared spectrometry (FTIR, Thermo Nicolet 6700, Thermo Fisher Scientific) at a resolution of 4 cm^−1^.

### 2.3. Soil Sample Collection

The test soil was collected from the farmland of Bu-mo village, Dongfang, Hainan. The surface layer at a depth of 0–20 cm was collected in the field, and after surface debris was removed, the soil was put into sterile self-sealing bags, placed in a constant-temperature sampling box (DNP-9082, Shanghai Jinghong Laboratory Equipment Co., Ltd., Shanghai, China), and transported to the laboratory refrigerator for storage at 4 °C. The soil was air-dried to remove plant and animal debris and sieved through a 0.25 mm sieve.

### 2.4. Biomass Preparation

The bagasse was dried at 60 °C for 12 h, then crushed and sieved through a 0.15 mm sieve, and stored for later use.2.5. Soil Incubation Experiments

Before the incubation experiments, the soil was incubated for two weeks to stabilize the soil microbial community. Fragments of biodegradable agricultural film from three different years (0.4% *w*/*w*) and ground sugarcane bagasse (0.4% *w*/*w*) were added to a 250 mL glass jar containing 100 g of sieved soil and were thoroughly mixed; the 0.4% *w*/*w* loading was chosen based on previous studies, which indicated that the concentration of agricultural film residues in field soils typically ranges from 0.1% to 0.5% [19,20,21]. A field water holding capacity of 70% was maintained for each sample, with CK serving as the blank control. Each sample was replicated three times. The samples were divided into five treatments: (1) CK: initial soil; (2) T1: biodegradable agro-film from 2022 mixed with soil; (3) T2: biodegradable agro-film from 2023 mixed with soil; (4) T3: biodegradable agro-film from 2024 mixed with soil; (5) T4: biomass mixed with soil (Table 1, Figure S1). The soil was incubated in the laboratory at 25 °C under dark conditions for 60 days to simulate the real soil environment of agricultural film residues. During the incubation period, ultrapure water was added every 7 days to maintain the field capacity (70%), and soil moisture was maintained weekly by weighing. At the end of the incubation, soil samples were collected, sieved through a 2 mm mesh, and stored at −80 °C in a laboratory freezer (DW-86L626, Haier, Qingdao, China) for subsequent measurement of soil enzyme activity.

### 2.5. Soil pH Determination

Soil pH was determined using a pH meter with a soil–water ratio of 1:5.

### 2.6. Measurement of Soil Enzyme Activity

Urease was measured by sodium hypochlorite–sodium phenol colorimetric assay, sucrase enzyme activity was measured by 3,5-dinitrosalicylic acid colorimetric assay, and alkaline phosphatase was measured by disodium phosphate colorimetric assay [22].

### 2.7. Soil DOM Extraction and Characterization

#### 2.7.1. Soil DOM Extraction

Soil DOM was extracted by the water–soil mixing and shaking method. An amount of 1.000 ± 0.0005 g of soil sieved through a 100-mesh sieve was dissolved in 10 mL of water, mixed thoroughly, and then shaken in a constant-temperature shaker (THZ-98A, Shanghai Yiheng Scientific Instruments Co., Ltd., Shanghai, China) at 200 rpm∙min^−1^ for 24 h at 25 °C; the mixture was protected from light, centrifuged at 5000 rpm∙min^−1^ for 10 min after shaking, and then filtered through a 0.45 μm filter membrane [23]. The clarified solution to be measured was further analyzed by a 3D fluorescence analyzer (F-380, Tianjin, China).

#### 2.7.2. Extraction of DOM from Mulch Leachate

For the extraction of DOM from the ground film, the biodegradable films of three years were cut into 1 × 1 cm pieces, mixed with deionized water at a solid–liquid ratio of 1:20, and then continuously shaken at 200 rpm∙min^−1^ in a constant-temperature shaker at 25 °C, protected from light, for 24 h. The shaken water–soil mixture was centrifuged at 5000 rpm∙min^−1^ for 10 min and then filtered through a 0.45 μm filter membrane. The clarified solution was further analyzed by a 3D fluorescence analyzer. In this study, three parameters, fluorescence index (FI), autochthonous index (BIX), and humification index (HIX), were used to characterize the nature of the source of DOM in PBAT + PLA-treated soils from different years [24]. The source of humus-like substances in DOM is indicated by the FI value: when the value is less than 1.4, it means that the DOM is an exogenous input; when it is greater than 1.9, it means that the DOM is mainly derived from microbial transformation products, and when the value is between 1.4 and 1.9, it means that DOM from both sources coexists [25]. The BIX is a measure of the proportion of its autochthonous components in DOM. The higher the value of BIX, the more prominent the autochthonous characteristics of DOM in soil. A value of BIX in the range of 0.6–0.7 usually implies that exogenous organic matter inputs have a significant effect on the composition of DOM; when the value of BIX is in the range of 0.8–1.0, the autochthonous contributions to DOM are more significant. When autochthonous source contribution is more significant, DOM in the soil is mainly produced by biosynthetic processes [26]. The degree of humification of DOM is generally assessed using the humification HIX index; when the value of HIX is less than 4, it indicates a low degree of DOM humification, while when the value of HIX is between 10 and 16, it indicates that DOM has significant humic characteristics.

#### 2.7.3. Fluorescent Composition Analysis of DOM

Excitation–emission matrix (EEM) fluorescence spectroscopy and PARAFAC (Parallel Factor Analysis) modeling were used to explore the fluorescence composition of soil and agricultural film DOM. The detailed method of operation was as follows. Fluorescence EEM was measured by three-dimensional fluorescence spectroscopy using a 150 W xenon arc lamp as a light source with a scanning speed of 1200 nm∙min^−1^. The excitation wavelengths were in the range of 200 nm to 550 nm, and the emission wavelengths ranged from 250 nm to 600 nm. Then, the DOM Fluor toolbox in MATLAB2018b was used with the PARAFAC model to analyze the EEM fluorescence spectral data. Before running the PARAFAC model, the data were preprocessed by blank subtraction and interpolation using the MATLAB program, and Raman and Rayleigh scattering in the EEM fluorescence spectra were automatically removed. The fluorescence data were used to divide soil DOM into five regions [27], as shown in Table 2.

The study employed three parameters (FI, BIX, and HIX) to characterize the source properties of soil DOM exposed to varying concentrations of plastic debris (Table 3).

### 2.8. Speciation Analysis of Heavy Metals in Soils

The Tessier extraction [28] method was used to continuously extract the heavy metals Cd, Zn, Mn, and Cu from the soil in the following forms: exchangeable (F1), carbonate-bound (F2), Fe- and Mn-oxide-bound (F3), organically bound (F4), and residual (F5); the concentrations of the metals in the soil samples extracted according to the above steps were determined by inductively coupled plasma mass spectrometry (ICP-MS, AFS-9800, PerkinElmer, Waltham, MA, USA), and included measurement of blank tests and soil standard samples (GBW(E)070235) [29]. Quality control was carried out using a blank test and a soil standard sample (GBW(E)070235) to ensure the accuracy of the results. The quality control analysis revealed that the measurement error was less than 5%. The recoveries were 95~101% (Table S1).

### 2.9. Data Processing

Data were processed in Excel2010 (Microsoft Corporation, Redmond, WA, USA) and analyzed by ANOVA using SPSS26 (IBM Corporation, Armonk, NY, USA); Raman scattering was eliminated by subtracting the 3D fluorescence spectral data of the blank ultrapure water before processing the DOM spectral data, and the Rayleigh-scattered regions in the DOM spectra were further corrected using MATLAB R2018b (MathWorks, Natick, MA, USA). The 3D fluorescence spectra maps and fluorescence region integrals were plotted and calculated by Origin2018 (OriginLab Corporation, Northampton, MA, USA).

## 3. Results and Discussion

### 3.1. Changes in Surface Morphology of Biodegradable Film Agro-Film of Different Vintages

**Figure 1:**
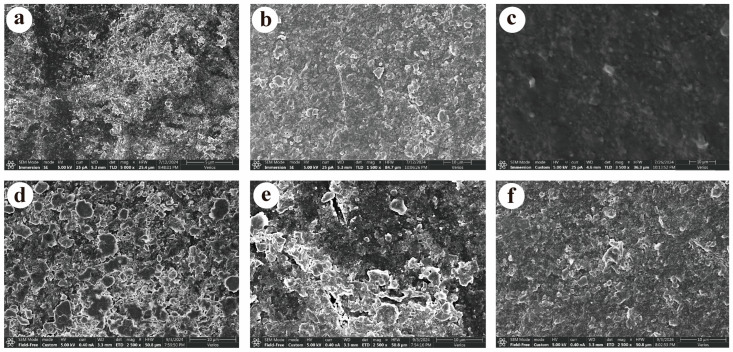
SEM of residual membranes from three different years before and after incubation experiments (×20,000 magnification). Notes: The surface morphology of the biodegradable agricultural films before and after the incubation experiment is shown in (**a**–**c**) and (**d**–**f**), respectively, corresponding to films aged for 2 years (T1), 1 year (T2), and new films (T3).

The surface characteristics of biodegradable agricultural films change significantly with aging, becoming more pronounced after two years. SEM analysis revealed that T3 initially had a relatively smooth surface with only a few bumps, while T2 showed roughness, wrinkles, and increased pits and particles. T1 exhibited the most severe fragmentation and structural disintegration, forming numerous small fragments (Figure 1a–c). After two months of soil incubation, these changes became more evident (Figure 1d–f), with aged films showing increased surface roughness and fragmentation [29]. This trend aligns with previous studies on PLA and PBAT plastics, where surface characteristics of biodegradable films in soil showed similar aging patterns [30]. In this study, T3 developed more noticeable bumps and increased surface roughness, while T2 and T1 showed more grooves, notches, and folds. Notably, T1 developed a “honeycomb”-like porous structure, indicating significant aging after one to two years of natural indoor storage.

### 3.2. FTIR Spectral Analysis of Biodegradable Agricultural Films from Three Different Years

**Figure 2:**
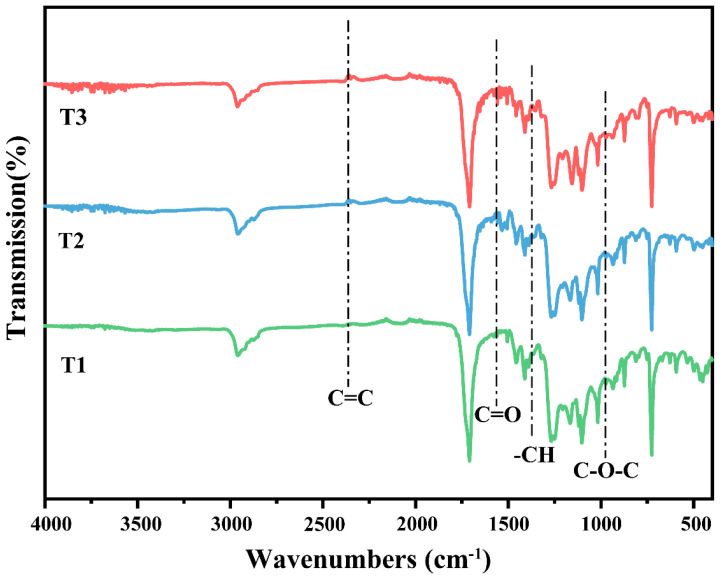
FTIR spectra of biodegradable mulch agricultural films from T1–T3 (T1: biodegradable agricultural film fragments in 2022, T2: biodegradable agricultural film fragments by 2023, T3: biodegradable agricultural film fragments by 2024).

FTIR spectroscopy revealed reductions in the intensity of specific functional groups in aged biodegradable agricultural films. Films from different years exhibited distinct spectral characteristics under natural aging conditions (Figure 2). The absorption peak intensity at 1368 cm^−1^, corresponding to C-H stretching vibrations, decreased gradually with film aging, a feature typical of aliphatic compounds [30]. The peak at 1563 cm^−1^, associated with C=O stretching vibrations, disappeared, likely due to the breakdown of aromatic benzene rings during aging [30]. The absorption peak at 2356 cm^−1^, attributed to C=C, was weaker in T1 than in T2, possibly due to environmental interference from atmospheric CO_2_ during FTIR analysis [13]. Additionally, the absorption peak at 1140 cm^−1^, linked to C-O-C stretching vibrations in ester or ether bonds (e.g., in PLA and PBAT), also decreased over time, indicating degradation of these bonds during aging [31].

### 3.3. DOM Spectral Analysis of Leachate from Biodegradable Agricultural Films from Three Different Years

**Figure 3:**
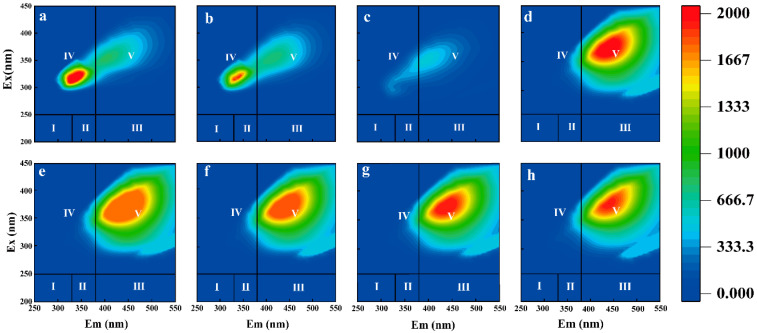
Fluorescence spectra of DOM (dissolved organic matter) leachate from biodegradable agricultural films in (**a**) T1, (**b**) T2, and (**c**) T3; fluorescence spectra of soil DOM (dissolved organic matter) under different treatments: biodegradable agricultural films in (**d**) T1, (**g**) T2, (**f**) T3, (**e**) CK, and (**h**) T4. Note: I: B peak (λex: 220–250 nm, λem: 280–330 nm)—Tyrosine-like substances. II: T peak (λex: 220–250 nm, λem: 330–380 nm)—Tryptophan-like substances. III: A peak (λex: 220–250 nm, λem: 380–480 nm)—Fulvic acids. IV: D peak (λex: 250–360 nm, λem: 280–380 nm)—Dissolved microbial by-products. V: C peak (λex: 250–420 nm, λem: 380–520 nm)—Humic acids.

The fluorescence intensity of DOM in the biodegradable agricultural film leachate increased gradually with the aging degree of the agricultural films. The DOM of the three biodegradable film agro-films showed different fluorescence intensities and characteristic peaks were observed in region IV (Ex/Em = 250–360 nm/280–380 nm dissolved microbial by-products) and region V (Ex/Em = 250–420 nm/380–520 nm humic acid) for the T1, T2, and T3 samples (Figure 3a–c). The characteristic peak corresponding to fluorescent substances similar to phenol was also present in microplastic leachate [32]. The appearance of such characteristic peaks in the IV and V regions may arise from the DOM in aged agro-film leachate, which mainly consists of phenolics (Ex/Em = 265–285/310–345 nm), and the distribution of proteins and phenolics in its DOM components is relatively high [33]. Consequently, T1 and T2 had higher fluorescence intensity than T3, which may be due to the release of polymer additives (e.g., plasticizers) or chain-break products from aged agricultural films, which caused enhanced fluorescence response [34].

### 3.4. Effect of Different Treatment Groups on Soil pH

**Figure 4:**
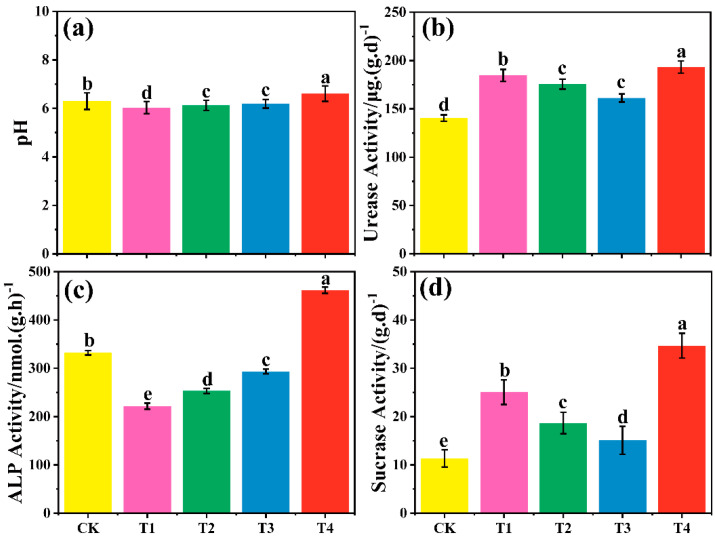
Changes in (**a**) soil pH and (**b**) activities of urease, (**c**) ALP: alkaline phosphatase, and (**d**) sucrase under different treatments. Note: Data points and error bars represent means ± S.E. (n = 3), According to Duncan’s test, different letters indicate significant differences (*p* < 0.05) among treatments.

Biodegradable agricultural films decreased soil pH, with a greater decrease observed as aging time increased, while biomass addition increased soil pH. Specifically, T1, T2, and T3 decreased soil pH by 4.28%, 2.83%, and 1.71%, respectively, with T1 showing the most significant reduction (Figure 4a, *p* < 0.05). This may be attributed to the release of acidic compounds during the degradation of biodegradable films, leading to soil acidification [35]. PLA, a polymer primarily synthesized from lactic acid, is likely a major contributor to soil acidification due to its acidic degradation products [36]. In contrast, T4 increased soil pH by 8.7% compared to CK, indicating that biomass addition can raise soil pH, which is opposite to the effect of aged biodegradable films [37,38].

### 3.5. Effect of Different Treatment Groups on Soil Enzyme Activities

Biodegradable agricultural film and biomass addition significantly increased soil urease activity (Figure 4b, *p* < 0.05). Compared with CK, the urease activity of T1, T2, T3, and T4 increased by 32.0%, 24.9%, 14.8%, and 40.3%, respectively. The urease activity of aged biodegradable films increases with the passage of aging time, which may be due to the enhanced stimulation of urease activity [39]. Shi et al. demonstrated that biodegradable microplastics significantly increased soil urease activity in line with this study [40,41]. In addition, the addition of biomass (T4) also enhanced urease activity, indicating that biomass has a positive impact on enzyme activity [42].

The impact of biodegradable agricultural films on soil alkaline phosphatase activity depends on the material type, specific surface area, and soil pH. Adding films of different ages reduced alkaline phosphatase activity (Figure 4c, *p* < 0.05). Compared to CK, T1, T2, and T3 showed decreases of 33.2%, 23.8%, and 11.6%, respectively, while T4 increased by 39.01%. Activity decreased with film aging, likely due to increased specific surface area as films degraded into smaller fragments [16,43]. These fragments release more toxic chemicals (e.g., phthalates) into the soil, inhibiting microbial alkaline phosphatase activity [44,45]. Conversely, biomass addition increased soil alkalinity and alkaline phosphatase activity by reducing soil acidity [46]. Lower soil pH may also inhibit alkaline phosphatase activity [47], affecting microbial communities associated with this enzyme.

The addition of T1–T4 significantly increased soil sucrase activity, with T4 showing the most significant improvement effect, increasing by 205.57% compared to the CK. For biodegradable films, T1 and T2 increased sucrase activity by 120.81% and 67%, respectively, while T3 increased by 32.95% (Figure 4d, *p* < 0.05). This suggests that the promotion of sucrase activity by aged films is positively correlated with aging time, likely due to increased carbon source availability [15]. Similarly, biomass addition (T4) significantly increased sucrase activity, consistent with previous findings that biomass enhances short-term sucrase activity [48]. These results highlight the role of aged films and biomass in stimulating sucrase activity by providing organic carbon substrates.

### 3.6. Effect of Different Treatment Groups on Soil DOM: Comparative Analysis with DOM Spectral Characteristics of Leachate

#### 3.6.1. Comparative Analysis of DOM Fluorescence Characteristics Between Leachate and Soil Systems

The fluorescence characteristics of DOM in aged biodegradable film leachate are similar and interactive with soil DOM under various treatments, indicating the transfer and transformation of DOM components from the film to the soil.

In the leachate study, the fluorescence intensity of DOM increased with film aging (T1 > T2 > T3), with distinct peaks in Region IV (Ex/Em = 250–360/280–380 nm, microbial by-products) and Region V (Ex/Em = 250–420/380–520 nm, humic acid-like substances). These peaks were attributed to phenolic compounds and protein-like substances released during film degradation, likely derived from polymer additives (e.g., plasticizers) and chain-break products [49]. Similarly, soil DOM treated with T1 and T2 showed enhanced fluorescence intensity in Region V (Figure 3d,g,h), aligning with the leachate results. The observed consistency indicates that aromatic and phenolic compounds released from aged films play a direct role in shaping the composition of soil DOM. Notably, the T4 also exhibited high fluorescence intensity in Region V. However, this phenomenon was associated with the promotion of soil humification through exogenous organic matter input, which fundamentally differs from the additive-induced DOM release observed in film treatments [50].

#### 3.6.2. Mechanism Linking Aged Films and Soil DOM Humification

The HIX of soil DOM increased with film age (T1: 9.20 ± 0.28; T2: 8.87 ± 0.33; T3: 8.26 ± 0.26), mirroring the elevated HIX observed in leachate DOM from aged films (Table 3). The observed correlation demonstrates that the aging process of biodegradable agricultural films facilitates the release of humic substances from the polymeric matrix into the soil environment, suggesting that the incorporation of biodegradable residues could potentially enhance soil humification processes [51]. The FI values of soil DOM (T1: 1.29 ± 0.03; T2: 1.33 ± 0.02; T3: 1.38 ± 0.03) decreased compared to CK (1.64 ± 0.05), confirming that film-derived DOM primarily originates from exogenous inputs (FI < 1.4). T4, as an exogenous organic matter input, did not significantly alter the humification pathway, as evidenced by its HIX value of 10.43 ± 0.39 (Table 4). These findings demonstrate that aged plastic films not only serve as a potential source of aromatic DOM but also act as catalysts promoting soil humification processes. Furthermore, biomass addition appears to enhance humification through microbially mediated mechanisms [17].

#### 3.6.3. Synergistic Effects of DOM and Heavy Metal Speciation

The increased humification of soil DOM correlates with reduced bioavailability of heavy metals. For example, the exchangeable Cd F1 decreased by 22.9% (T1), 13.1% (T2), and 10.2% (T3), while F5 increased by 59.6%, 50.2%, and 36.1%, respectively. The observed phenomenon corresponds with the elevated HIX values in aged film treatments, given that humified DOM is characterized by abundant oxygen-containing functional groups (e.g., carboxyl and phenolic hydroxyl), which facilitate complexation with heavy metals and promote their stabilization in F4 and F5. A similar mechanism was observed in the filtrate DOM, where aged films released aromatic structures of benzene rings and aliphatic compounds capable of binding to metals (FTIR peaks located at 1563 cm^−1^ and 1368 cm^−1^). The findings reveal that aged films not only alter the composition of soil DOM but also indirectly modulate heavy metal speciation through DOM-mediated complexation processes.

### 3.7. Speciation Analysis of Soil Heavy Metals in Different Treatment Groups

**Figure 5:**
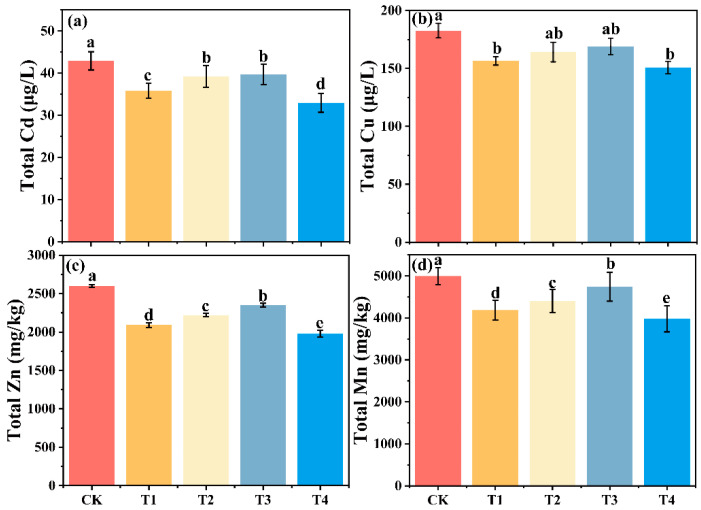
Changes in the total amounts of Cd, Cu, Zn, and Mn in the soil in different treatment groups. Note: (**a**) Cd (Cadmium), (**b**) Cu (Copper), (**c**) Zn (Zinc), and (**d**) Mn (Manganese) total content. Data points and error bars represent means ± S.E. According to Duncan’s test, different letters indicate significant differences (*p* < 0.05) among treatments.

Biodegradable films of different age groups promote the transformation of Cd, Zn, Cu, and Mn from F1 to F4 and F5, which may be driven by the increase in soil DOM and oxygen-containing functional groups. Specifically, Cd is mainly present in F1, Zn and Mn are present in F5, and Cu is present in F4 (Figure 5).

**Figure 6:**
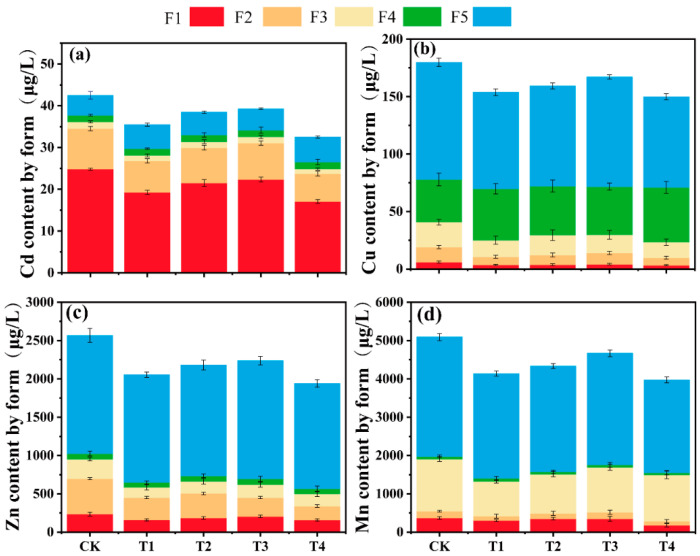
Changes in the chemical speciation ratios of Cd, Cu, Zn, and Mn in the soil in different treatment groups. Note: (**a**) Cd (Cadmium), (**b**) Cu (Copper), (**c**) Zn (Zinc), and (**d**) Mn (Manganese). Speciation: exchangeable (F1), carbonate-bound (F2), Fe- and Mn-oxide-bound (F3), organically bound (F4), and residual (F5). Data points and error bars represent means ± S.E. (n = 3).

For Cd, compared to CK, the proportion of F1 decreased by 22.9%, 13.1%, and 10.2% in T1–T3, respectively, while F5 increased by 59.6%, 50.2%, and 36.1%, respectively. In T4, F1 decreased by 21.9% and F5 increased by 65.1%. Additionally, the proportions of F2 and F3 decreased slightly with the aging of the biodegradable agricultural film (Figure 6a); for Cu, F4 and F5 were generally higher across all treatments. Compared to CK, F4 increased by 20.7%, 15.2%, and 13.4% in T1–T3, respectively, while F5 decreased by 17.7%, 14.6%, 6.4%, and 23.5% in T1–T4, respectively (Figure 6b). For Zn, compared to CK, the proportion of F1 decreased by 31.5%, 21.7%, and 12.8% in T1–T3, respectively, with T4 showing the largest reduction at 33.3%. F2 also decreased, while F3 remained relatively stable. In contrast, F5 decreased by 20.7%, 14.9%, and 12.7% in T1–T3, respectively (Figure 6c). For Mn, T1–T3 showed decreased F1 and F2 fractions compared to CK, though not statistically significant. In contrast, F3 decreased significantly with film age, by 29.1%, 25.3%, and 18.8% in T1–T3, respectively (Figure 6d).

Aged biodegradable agro-film addition promotes the conversion of Cu and Zn from bioavailable forms to stable organically bound and residual forms [52]. For Cd, Cu, Zn, and Mn, the proportions of F1, F2, and F3 decreased with increasing film aging time. This may be due to the presence of DOM in the soil, which contains abundant functional groups (e.g., amides, carboxyls, phenolic hydroxyls) that readily form complexes with heavy metals, thereby immobilizing them [53,54]. Additionally, aging of biodegradable film debris increases the abundance of oxygen-containing functional groups, which further enhance heavy metal adsorption and reduce their bioavailability [55]. For Cu, high organic binding affinity is attributed to its strong interaction with carboxyl and aromatic groups in DOM [56]. SEM analysis also revealed that aged film fragments have increased surface area due to cracks and cavities, which can adsorb heavy metals and reduce their bioavailability [56]. Thus, the bioavailability of heavy metals in soil is influenced by both the aging of biodegradable film fragments and their interactions with soil organic matter.

### 3.8. Correlation Analysis

The HIX index of soil DOM humification is significantly negatively correlated with total Cd (*r* = −0.84) and bioavailable Cu (F1 + F2) content (*r* = −0.88), suggesting that enhanced humification reduces heavy metal fractions. This may be due to aged biodegradable films releasing more DOM and oxygen-containing functional groups, which improve soil humification and heavy metal adsorption capacity, thereby reducing bioavailability [18]. Additionally, biomass addition increases soil organic matter content and promotes heavy metal adsorption, as shown in studies using sugarcane bagasse and straw [18].

Soil urease activity is significantly negatively correlated with bioavailable Cu (F1 + F2) and total Cu content (*r* = −0.74 and *r* = −0.78, respectively). This is attributed to ligand reactions causing structural changes in urease active sites, making it a potential bioindicator of soil health under heavy metal stress [57].

**Figure 7:**
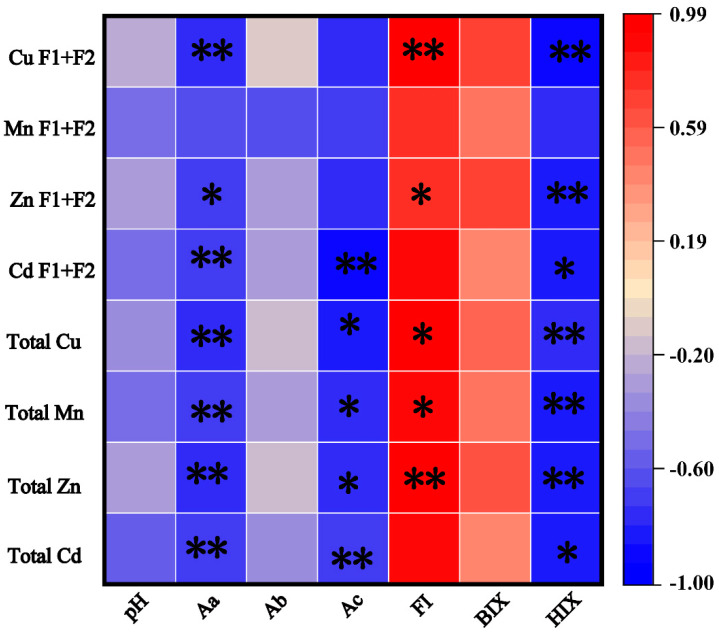
Correlation analysis between soil pH, DOM, and the total and available concentrations of heavy metals among different treatment groups. (Aa: urease activity, Ab: alkaline phosphatase activity, Ac: sucrase activity, FI (fluorescence index), BIX (autochthonous index), HIX (humification index), F1 (Exchangeable fraction), F2 (Carbonate-bound fraction), Cd (cadmium), Cu (copper), Zn (zinc), and Mn (manganese)). Note: Red bars in the color column indicate positive correlations, and blue bars indicate negative correlations; the deeper the color, the stronger the correlation. * denotes *p* ≤ 0.05; ** denotes *p* ≤ 0.01.

Soil pH affects heavy metal reactivity by altering adsorption sites and chemical states. Although no significant correlation was found between pH and bioavailable or total heavy metals (*r* = −0.24 and *r* = −0.34, respectively), pH still regulates metal bioavailability (Figure 7). Lower pH enhances microplastic adsorption of heavy metals but reduces clay mineral adsorption capacity due to increased H+ competition, increasing heavy metal mobility [55,58]. Fluctuations in soil pH have a significant effect on the adsorption behavior of heavy metals, which is regulated by a combination of heavy metal species and environmental conditions [59,60]. The accumulation and transport behaviors of the heavy metals Cd, Cu, Zn, and Mn in aged biodegradable agricultural film fragments in soil are primarily influenced by soil pH and DOM. Therefore, systematically elucidating the interaction mechanisms between biodegradable film residues, soil pH, and DOM can provide a new theoretical perspective for in-depth understanding of the adsorption behavior, migration and transformation processes, and ecological risks of heavy metals in soil mediated by biodegradable film residues. This is particularly significant in the context of DOM-driven humification processes (e.g., surface complexation, ion exchange) and pH-regulated charge effects.

## 4. Conclusions

This study investigated the effects of aged PBAT + PLA biodegradable agricultural film fragments on soil properties and heavy metal speciation. Aging led to surface roughening, fragmentation, and reduced infrared absorption peaks (C-H, C=O, C-O-C, and C=C), indicating degradation. Aged films released DOM with higher fluorescence intensity, enhancing soil humification and promoting the complexation of heavy metals, thereby reducing their bioavailability. Soil pH decreased with film aging due to organic acid release, while biomass addition increased pH. Aged films increased urease and sucrase activities but decreased alkaline phosphatase activity, likely due to toxicity from increased surface area.

Heavy metal speciation analysis revealed that aged films reduced exchangeable, carbonate-bound, and iron–manganese oxide-bound fractions while increasing organically bound and residual fractions, particularly for Cu. This transformation was driven by enhanced DOM humification and oxygen-containing functional groups, which stabilized heavy metals in less bioavailable forms. Correlation analyses showed that soil humification negatively correlated with heavy metal bioavailability, while soil pH played a role in modulating metal adsorption and desorption.

In summary, aged biodegradable agricultural films can affect soil physicochemical properties, alter enzyme activity, and influence heavy metal dynamics by promoting the release of DOM, thereby reducing the bioavailability of metals. However, the long-term ecological impacts, particularly on soil microorganisms and plant growth, require further investigation to assess the sustainability of biodegradable films in agriculture.

## Figures and Tables

**Table 1:** Experiment number configuration.

Experimental Sample	Treatment Number
Initial soil	CK
Add 2022 residual film 0.4% (*w*/*w*)	T1
Add 2023 residual film 0.4% (*w*/*w*)	T2
Add 2024 residual film 0.4% (*w*/*w*)	T3
Add biomass 0.4% (*w*/*w*)	T4

**Table 2:** Range of fluorophore (peak) region integration.

Region	Fluorescence Peak	Fluorescence Integral Region	Type of Fluorescent Substance
I	B	λex: 220–250 nm, λem: 280–330 nm	Tyrosine-like
II	T	λex: 220–250 nm, λem: 330–380 nm	Tryptophan-like
III	A	λex: 220–250 nm, λem: 380–480 nm	Fulvic acids
IV	D	λex: 250–360 nm, λem: 280–380 nm	Dissolved microbial by-products
V	C	λex: 250–420 nm, λem: 380–520 nm	Humic acids

**Table 3:** Description of fluorescence spectral parameters.

Fluorescence Spectral Parameters	Definition	Description
FI	The ratio of fluorescence emission spectral intensity at 450 nm to 500 nm for an excitation wavelength of 370 nm	Humus-like sources in DOM can be characterized, where FI > 1.9 indicates that DOM is mainly derived from microbial activities, and FI < 1.4 denotes DOM is mainly derived from terrestrial plants and soil organic matter, which are exogenous inputs
BIX	The ratio of fluorescence emission spectral intensity at 380 nm to 430 nm for an excitation wavelength of 310 nm	BIX > 1 means DOM mainly caused by organisms or bacteria; BIX 0.6–0.7 means DOM imported by terrestrial sources or strongly influenced by humans
HIX	The ratio of the spectral area in the fluorescence emission spectrum with emission wavelengths in the band of 435–480 nm to 300–345 nm at an excitation wavelength of 254 nm	HIX characterizes the degree of humification or maturity of DOM; HIX < 4 indicates that DOM is weakly humified, and HIX between 10 and 16 indicates that DOM has significant humus characteristics

Note: fluorescence index (FI), autochthonous index (BIX), and humification index (HIX).

**Table 4:** Description of fluorescence spectrum parameters.

Treatment	FI	BIX	HIX
CK	1.64 ± 0.05 a	0.82 ± 0.01 a	5.53 ± 0.06 c
T1	1.29 ± 0.03 b	0.60 ± 0.02 bc	9.20 ± 0.28 b
T2	1.33 ± 0.02 b	0.58 ± 0.04 bc	8.87 ± 0.33 b
T3	1.38 ± 0.03 b	0.54 ± 0.02 c	8.26 ± 0.26 b
T4	1.28 ± 0.03 b	0.65 ± 0.06 b	10.43 ± 0.39 a

Note: According to Duncan’s test, different lowercase letters in the same column indicate significant differences between treatments (*p* < 0.05). The data points and error bars represent the mean ± standard error (n = 3). FI (fluorescence index), BIX (autochthonous index), HIX (humification index).

## Data Availability

The data that support the findings of this study are available from the corresponding author upon reasonable request.

## Supplementary Materials

The following supporting information can be downloaded at: https://www.mdpi.com/article/10.3390/toxics13040245/s1, Figure S1: Experimental process diagrams; Table S1: The content of different forms of heavy metal elements in different treatment groups.
