# Supplementary material for: Effects of Aging Biodegradable Agricultural Films on Soil Physicochemical Properties and Heavy Metal Speciation
Source: Toxics. 2025 Mar 26;13(4):245. doi: 10.3390/toxics13040245 (PMC12030900; doi:10.3390/toxics13040245)
Supplement: Supplementary file 1 [file toxics-13-00245-s001.zip › toxics-3509807-supplementary.pdf]

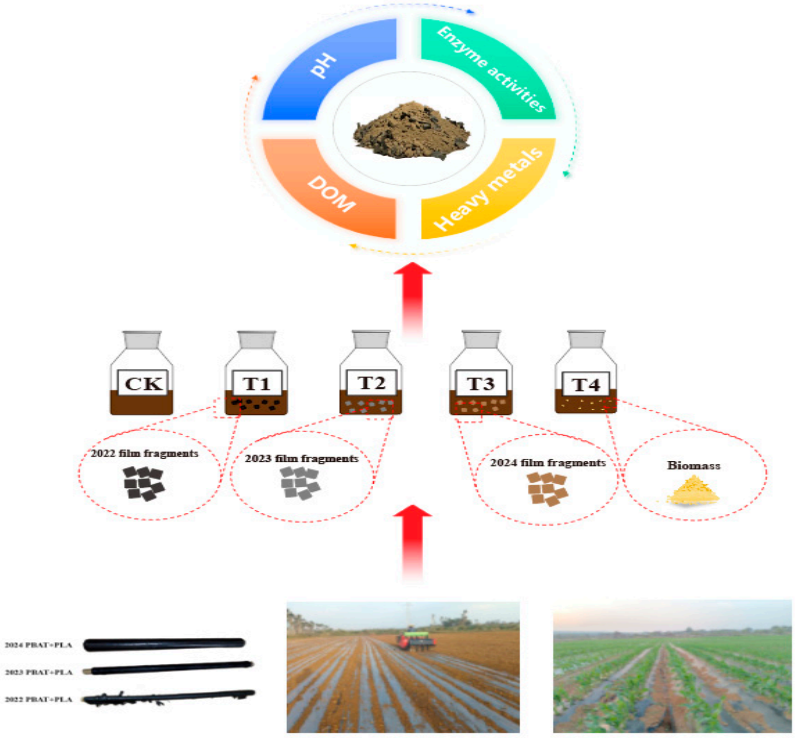

Figure S1 Experimental process diagrams.

Table S1 The content of different forms of heavy metal elements in different treatment groups.

Sample	Fraction	Cd (μg/L)	Cu(μg/L)	Zn (mg/kg)	Mn (mg/kg)
**CK**	F1	24.80±0.26	5.97 ± 0.86	235.86 ± 25.60	369.22 ± 32.60
	F2	9.71±0.52	13.06 ± 1.52	462.59 ± 14.20	176.87 ± 21.00
	F3	1.64±0.25	21.69 ± 2.50	253.51 ± 25.00	1362.06 ± 65.00
	F4	1.58±0.23	37.12 ± 5.60	74.12 ± 33.00	69.08 ± 36.00
	F5	4.79±0.91	102.00 ± 3.52	1540.28 ± 91.00	3114.55 ± 91.00
	Bulk analysis	42.93 ± 2.17	182.56 ± 14.00	2599.46 ± 188.80	4996.13 ± 245.60
	Recovery (%)	99.08	99.96	98.72	101.91
**T1**	F1	19.27 ± 0.47	3.50 ± 0.47	161.42 ± 14.60	301.45 ± 25.00
	F2	7.54 ± 0.51	7.05 ± 1.36	289.96 ± 15.50	115.44 ± 51.00
	F3	1.28 ± 0.35	14.44 ± 3.50	133.37 ± 36.00	906.01 ± 45.00
	F4	1.63 ± 0.14	44.80 ± 4.60	64.83 ± 21.00	81.04 ± 48.00
	F5	5.75 ± 0.35	84.02 ± 3.00	1403.50 ± 35.00	2734.67 ± 66.00
	Bulk analysis	35.85 ± 1.82	156.46 ± 12.93	2090.88 ± 122.10	4184.21 ± 235.00

	Recovery (%)	98.96	98.19	98.19	98.91
**T2**	F1	21.53 ± 0.78	3.74 ± 0.78	184.63 ± 17.80	349.08 ± 36.00
	F2	8.43 ± 0.56	8.42 ± 1.65	319.52 ± 16.50	138.96 ± 56.00
	F3	1.43 ± 0.46	17.26 ± 4.60	159.45 ± 37.00	1023.79 ± 68.00
	F4	1.63 ± 0.53	42.77 ± 5.20	71.18 ± 23.00	60.48 ± 47.00
	F5	5.46 ± 0.25	87.16 ± 2.50	1446.61 ± 65.00	2761.39 ± 65.00
	Bulk analysis	39.22 ± 2.58	164.12 ± 14.73	2221.05 ± 159.30	4402.31 ± 272.00
	Recovery (%)	98.1	97.08	98.21	98.44
**T3**	F1	22.33 ± 0.59	4.14 ± 0.59	205.58 ± 15.60	349.08 ± 65.00
	F2	8.74 ± 0.53	9.82 ± 1.32	245.42 ± 15.60	172.32 ± 56.00
	F3	1.48 ± 0.38	15.78 ± 3.80	168.37 ± 32.00	1169.81 ± 78.00
	F4	1.61 ± 0.74	41.95 ± 3.20	75.10 ± 35.00	66.12 ± 62.00
	F5	5.12 ± 0.19	95.42 ± 1.80	1543.92 ± 55.00	2908.90 ± 85.00
	Bulk analysis	4742.98 ± 346.00	168.95 ± 10.71	2350.82 ± 153.20	4742.98 ± 346.00
	Recovery (%)	98.38	95.26	95.22	98.38
**T4**	F1	17.07 ± 0.46	3.30 ± 0.46	157.86 ± 14.60	176.63 ± 45.00
	F2	6.68 ± 0.54	6.49 ± 1.23	181.74 ± 18.20	107.67 ± 42.20
	F3	1.13 ± 0.27	13.50 ± 2.70	160.83 ± 35.00	1207.61 ± 98.00
	F4	1.67 ± 0.67	47.77 ± 5.20	65.96 ± 35.00	59.45 ± 54.00
	F5	5.97 ± 0.27	78.83 ± 2.60	1374.02 ± 48.00	2418.69 ± 75.00
	Bulk analysis	32.95 ± 2.21	150.63 ± 12.19	1978.10 ± 150.80	3979.85 ± 314.20
	Recovery (%)	98.72	98.13	98.14	99.75

Notes: Recovery (%): The data of the sum of five fractions divided by bulk analysis result. The Bulk analysis corresponds to Figure 5 in the paper.
